# Transiliac lengthening with posterior lumbar-iliac percutaneous fusion in sacral hemiagenesis

**DOI:** 10.1007/s11751-011-0109-0

**Published:** 2011-07-22

**Authors:** Pedro Antonio Sánchez Mesa

**Affiliations:** Specialized Unit in Orthopedics and Traumatology S.A.S Club Hip & Knee, Bogotá, DC Colombia

**Keywords:** Postural imbalance, Hemiagenesis, Rotoscoliosis, Pelvis, Hemisacrum

## Abstract

Sacral agenesis is a term that applies to a wide range of developmental disorders of the lower portions of the spine and pelvis. Hemisacrum patients with all sacral segments present on one side of the spine, and decompensated lumbar rotoscoliosis, whit instability torac-pelvic that had transiliac lengthening of the lower extremity, accomplished by an innominate osteotomy with interposition of a rectangular iliac-bone graft in the osteotomy site, besides a posterior lumbar-iliac percutaneous fusion. We reported 5 adolescent patients, 2 men and 3 women, treated from 2000 to 2009, associated with average pelvic imbalance of 3.2 cm (2.5–4.5 cm) without other associated congenital anomalies. Patients classified as Vergara (Acta Ortop Mex 19:6–12, 2005) type IB unilateral partial agenesis of the sacrum, asymmetry of the pelvic ring there’s a torac-pelvic cifoscoliotic deformity. Mean age was 12.2 years-old (range from 8.2 to 13.7). The mean follow-up was 7.2 years (from 2 to 8). The consolidation process of the osteotomy site was in an average of 6.4 (5–8.7 weeks) (*P* = 0.036). None of the patients presented family medical history of diabetes on their mothers. None residual femoral nerve palsy. The procedure offers postural correction at the level of the pelvis, low morbidity and no additional operations were required to achieve the surgical objective. *Level of evidence* Level IV, therapeutic study: Case series (no, or historical, control group), Prospective: The study was started before the first patient was enrolled.

## Introduction

Sacral agenesis is a term that indicates that some portion of the lumbar spine, sacrum, or pelvis is incompletely or incorrectly formed at the time of birth, these are structures malformations derived from the caudal region of the embryo, such as, the urogenital system, the hindgut, caudal spine and spinal cord, and the lower limbs [1–4].

The etiology, classifications, and clinical features of sacral agenesis are discussed. Hohl in 1950 describes the first case of sacral agenesis, from that time isolated cases of this illness were described, in 1978 Renshaw [5] suggests a classification according to the radiological aspect of the lumbosacral agenesis, in 2002 Guille [6] suggests a new classification based on the relation of the sacral agenesis type and the children ambulatory function, perhaps, in these classifications, because the first one doesn’t give a forecast value on the instability grade of the lumbosacral spine and the ambulatory function, and the second one because of its complexity motivate us to use a classification that brings a topographic description, and a forecast value of the ambulatory function, as it’s described by the author. Vergara [7] sacral agenesis classification: *Type I.* Unilateral partial agenesis of the sacrum. *A. Stable:* despite the asymmetry of the pelvic ring there’s no progression on the torac-pelvic cifoscoliotic deformity. *B. Unstable:* when because of the asymmetry of the pelvic ring there’s a torac-pelvic cifoscoliotic deformity. Besides in each subtype is placed if it’s right or left, example: Type I–B-Left. *Type II.* Total sacral agenesis can or can’t be fused ilia or and iliac amphiarthrosis of the lowest vertebra. *A. Stable:* There’s no translation of the spine over the iliacs neither the torac-pelvic cifoscoliotic deformity. *B. Unestable:* when there’s translation or torac-pelvic cifoscoliotic deformity, that doesn’t allowed to be standing up without hands support. *Note*: Next to the subtype the lower vertebra has to be placed, even if it’s complete or a trace. Example: Type II-A-L3.

## Materials and methods

**Fig. 1:**
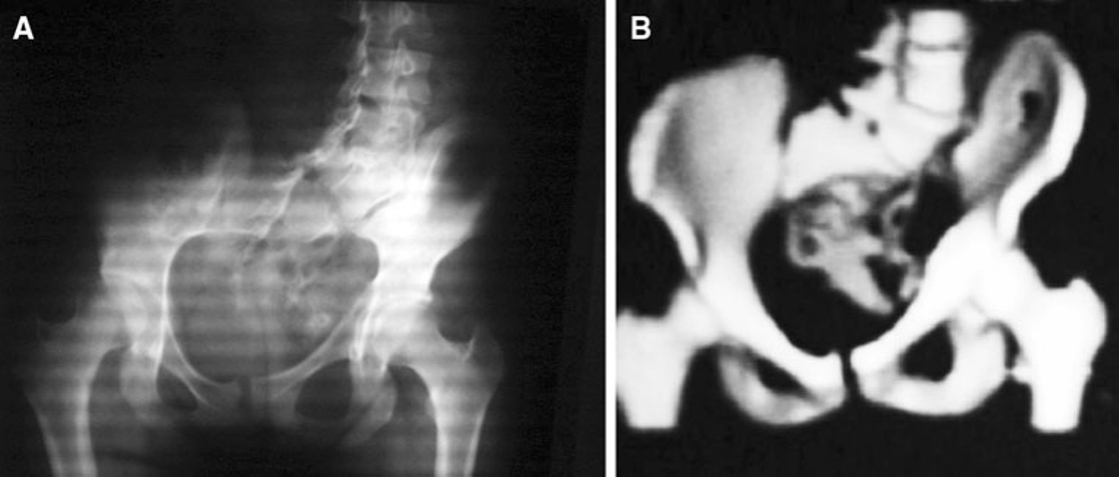
**a** A pelvic antero-posterior roentgenogram and a **b**, computed tomography scan of the pelvis with three-dimensional reconstruction revealed hemisacrum

**Fig. 2:**
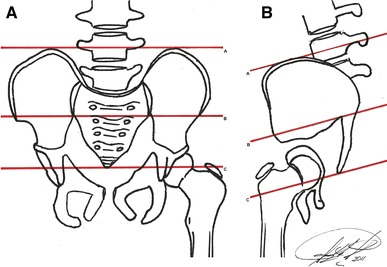
Sacral ratio index (SR). The *lines* are draw: the *A horizontal line* following the superior higher edge of the iliac bone; the *line B*, at the level of both post-inferiors iliacs spines, in inferior (most caudal) point at the left and right sacroiliac joints, and the *line C*, parallel to the previous on the more distal point of the visible coccyx (4 vertebrae), in the AP and lateral radiograph. The normal average of 0.7–0.8 (sacral ratio = BC/AB)

**Fig. 3:**
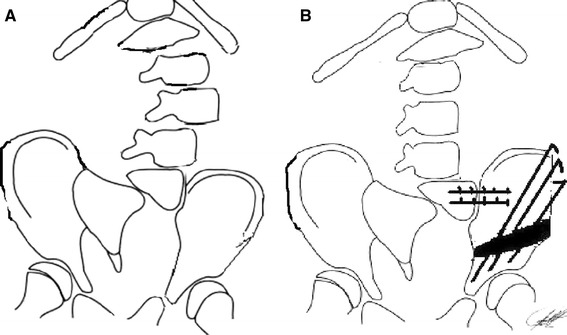
**a** Schematic illustrations show the initial deformity type IB left and **b**. Hemipelvis lengthening with rectangular bone graft and posterior lumbar-iliac fusion in sacral hemiagenesis

This is a case series of 5 patients who were treated between January 2000 to September 2009 at the Paediatric Orthopaedic Department. Patients who presented hemisacrum, a decompensated lumbar ciforotoscoliosis associated with a pelvic imbalance, and March with orthesis see Figs. 1, 2, 3, besides theses presented a mild urinary incontinence which may be associated with partial loss of motor and sensory function of the S2–S4 roots confirmed by cystoscopy and retrograde pyelography, with mild weakness of toe flexion, without constipation, neither other associated congenital anomalies with normal parents, without significant medical history, besides a neurological examination including peri-anal sensation and anal sphincter tone. A full blood count and serum urea, electrolyte and creatinine values were normal. The principal motive of consultation was their pelvic disbalance and shortening of the limb. In the radiographs images of the sacrum for this study were analyzed by the index called “Sacral Ratio” (SR) [8], making an index that takes into consideration the two measuring after tracing 3 horizontal lines in the radiographies: the A horizontal line following the superior higher edge of the iliac bone; the line B, at the level of both post-inferiors iliacs spines, in inferior (most caudal) point at the left and right sacroiliac joints, and the line C, parallel to the previous on the more distal point of the visible coccyx (4 vertebrae), in the AP and lateral radiograph. The SR is the percentage between the distances of the B and C lines; and from A to B. The normal average of 0.7–0.8 (sacral ratio = BC/AB). See Fig. 2 Anterior–posterior (a) and lateral (b) sacral ratios. The scanogram was used to measure limb-length discrepancy and the use of a full-length standing anteroposterior radiograph of the lower extremities and a growth study, consisting of serial orthoroentgenograms and bone—age determinations, was carried out in all patients with limb—length inequality center—edge angles were measured from roentgenograms made with the patient standing.

### Surgical technical

**Fig. 4:**
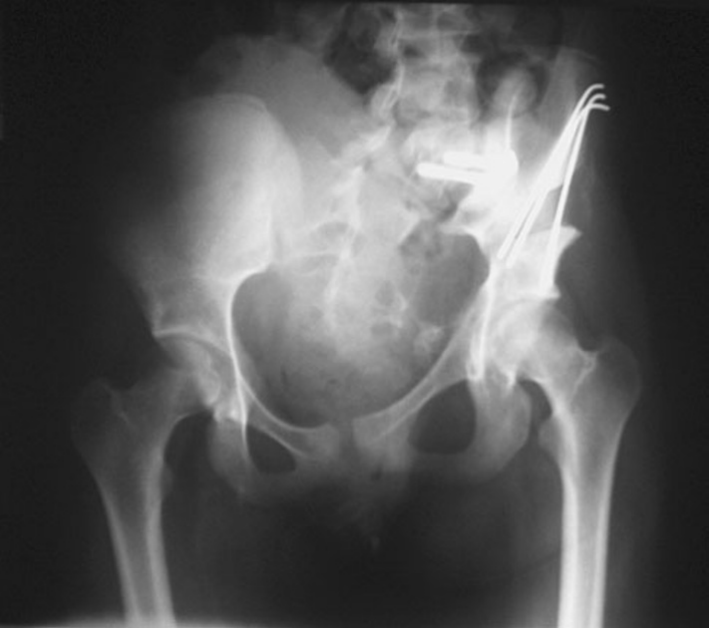
Anterior-posterior pelvis x-ray image shows a transiliac limb lengthening osteotomy using a interposition rectangular bone graft instead of the usual rectangular graft to achieve up to 4 cm of intrapelvic lengthening. It allows correction of certain forms of postural imbalance and pelvic obliquity, as well as allowing an optimal and variable amount of acetabular redirection

**Fig. 5:**
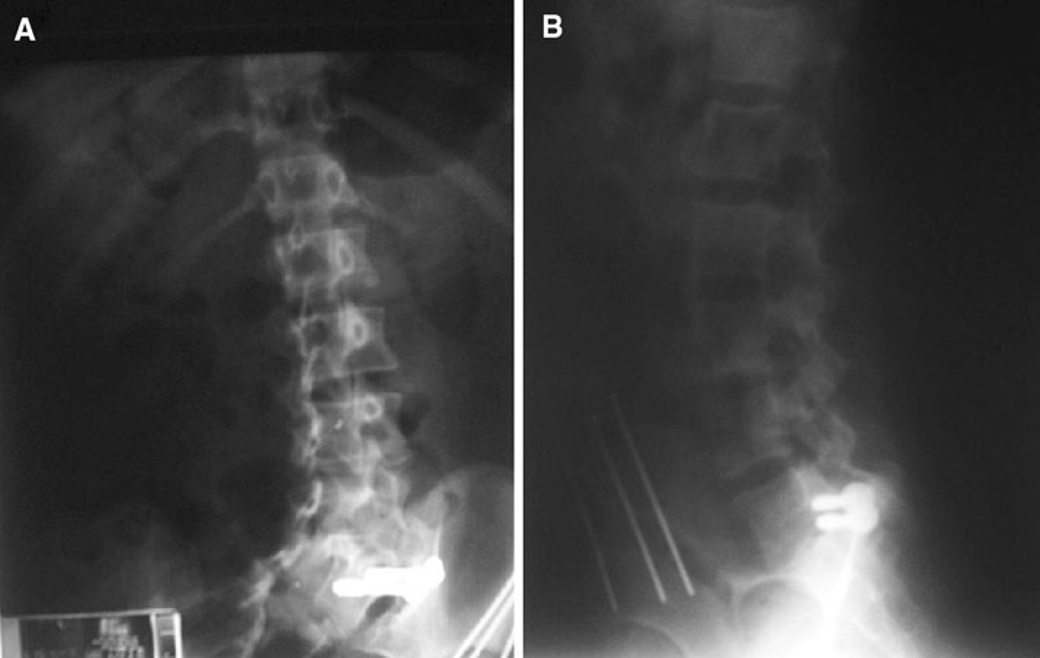
**a** and **b**. Posterior lumbo-pelvic fusion by percutaneous CT-guided application of screws

*First time*: The patients are placed in a prone position on a fluoro table; whose were made in a percutaneous way, a posterior lumbar-iliac fusion, executed under fluoroscopic control and CT guidance, through two cannulated screws of 6.5 mm, performing an arthrodesis of the L5 vertebral body with the iliac wing, see Figs. 3, 4, 5.

*In a second time*, but in the same surgical act, patients in the supine position; the surgical approach used is the same as that described by Salter [9–14] for innominate osteotomy for congenital dislocation of the hip. Preliminary tenotomies percutaneus of the adductors are made by an inguinal approach. By the Smith Peterson approach is performed and with the help of a Gigli saw osteotomized the innominate bone from the sciatic notch to the anterior inferior iliac spine. At this point, this technique is like the classic innominate osteotomy for congenital dislocation. The redirection of the acetabulum is performed associated to an intrapelvic distraction that is carried out with a laminar spreader and in this way the osteotomy is open, producing a lengthening of the hemipelvism, correcting the intrapelvic disbalance. The bone graft is wedged carefully into the distracted osteotomy site, and the laminar spreader is gently removed. A rectangular bone graft is then inserted into the osteotomy to maintain the desired acetabular redirection and the transiliac lengthening expected of the lower extremity see Fig. 3. The distracting force is directed to caudal, and distal, hinging occurs through the symphysis. Before is mandatory iliopsoas total tenotomy. The effective lengthening achieved will be equal to the height of the interposed graft directly superior to the hip joint. The sharp edge of ilium at the postero-superior corner of the bone graft site is then trimmed to allow reapproximation of the iliac apophysis during closure. The bone graft should remain solidly in place, stable under compression. At least two Steinmann pins has be drilled in parallel, beginning in the anterior portion of the proximal fragment, directed toward posterior and distally, transfixing both osteotomy fragments and the graft in their course posterior to the hip joint. The hip is then carried gently through its range of motion to rule out penetration other joint. It’s closed by classic technique of pelvic osteotomy described by Salter, Millis and Hall.

*Note*: these two procedures were performed during two-stage in a combined surgery.

## Results

We reviewed our experience with a transiliac lengthening osteotomy for sacral hemiagenesis with pelvic obliquity result intrapelvic bone asymmetry, associated with a posterior lumbar-iliac percutaneous fusion in all the patients. Their range ages at surgery were from 8.2 to 13.7 years (average 12.2). The mean follow-up was 7.2 years (2–8). The last patient was intervened 2 years before the end of the study. Postural balance was obtained and an intrapelvic symmetry acceptable post-correction of the deformity. They had an oblique lumbosacral joint and resultant lumbar rotoscoliosis. Average curve postoperative was 12.6 (8–16 degrees). The reduction averaged obtained in Cobb angle was 5 (4–8 degrees) (*P* = 0.041). In neither adolescent did the scoliotic curve progress in seventy nor did it require posterior spinal fusion with rods instrumentation. They all had type IB (Vergara), type IB left 4 patients and type IB right 1 patient with an average preoperative index SR was 0.55 (0.43–0.62) (*P* = 0.02), average 3 months postop SR index was 0.83 (0.76–0.93), average 24 months postop SR index was 0.76 (0.72–0.8). The osteotomy site consolidation process was 6 weeks confirmed by X-ray in average (from 5 to 8), (*P* = 0.036). Average pre-op pelvic imbalance was 4.1 (3.8–4.4 cm), (*P* = 0.029). Is has been observed that the patients with SR whose numbers are lower than 0.55 are related to a poor functional forecast, in the urogenital dysfunction and motion pattern see Fig. 4 and Table 1. The average residual limb length discrepancy after the procedure to 5 years follow-up was 1.1 cm (range 0.8–1.4 cm). All patients rapidly regained their preoperative ranges of hip motion. None residual femoral nerve palsy. One patient of 12-year-old boy subsequently underwent limb lengthening with external fixation see Fig. 2. Neither patient had dysplasia of the hip or contracture of the knee. The patients were community ambulators. All the girls presented their first menstrual period before consulting the orthopedic service. Two patient girls of the five patients with acetabular dysplasia and limb-length inequality achieved a balanced stance without a lift after surgery. All patients were associated with an increase in the center edge angle. Average center-edge (CE) angle pre-operative was 13.6 (11–16 degrees), average CE postoperative 25 (22–28 degrees), average acetabular angle preop. 39.6 (38–43 degrees) and postop. 20 (16–22 degrees).

**Table 1:** Includes patient’s demographics, functional impairments, walking ability, type of treatment, and pre- and postoperative radiographic measurements

Sex	Age	SR Index Pre-op	SR Index Post-op (24 months)	Pelvic Imbalance Pre-op (cms)	Limb length discrepancy (after 7 years follow-up) (cms)
Boy	12.8	0.56	0.76	3.9	1.1
Boy	13.7	0.62	0.8	3.8	0.8
Girl	8.2	0.43	0.72	4.4	1.4
Girl	13.6	0.58	0.76	4.2	1.2
Girl	12.9	0.56	0.78	4.2	1

### Postoperative care

Isometric exercises are begun immediately, mobilization in bloc of patients and bed rest. Gentle, active assisted exercises are begun on the third day. Roentgenograms are made at the first and third month, to check position and healing and taken during the periods of 1–2 and 4 years. The pins are removed at 12 months after the osteotomy.

### Complications

There were one wound infections. One patient had a superficial staphylococcus aureus infection none requiring incision to drainage neither intrasurgical washes, without hematomas, which were resolved with following intravenous first-generation cephalosporins therapy during 3 days of hospital stance, after oral manage in a 10 days treatment.

## Discussion

Transiliac Iimb-lengthening osteotomy, a modification of Salter’s innominate osteotomy is a useful method in treating postural imbalance of certain types of sacral agenesis, like an unusual congenital anomaly, requiring an early diagnosis. In many studies published in a global literature without a language restriction, these cases are reported as very rare, of sacral angenesis type IB, unestable by the asymmetry of the pelvic ring and torac-pelvic cifo-rotoscoliotic deformity.

Transiliac lengthening osteotomy with osteotomy posterior lumbar-iliac percutaneous fusion provides both leveling of the pelvis restoring postural balance [9–14]. The technique is relatively simple, occasionally; however, the degree of gait disturbance and the posture demands make the treatment even more aggressive. In these cases, our therapeutic program has included a Salter-type innominate osteotomy to lengthen the hemipelvis on the functionally low side. A rectangular bone graft is interposed in the osteotomy site, but bigger size and larger vertex than the original bone block described by Salter.

The purpose of this surgical technique is the correction of the postural imbalance secondary to the intrapelvic bone asymmetry, balancing this way, the lumbosacral junction and eliminates any postural secondary lumbar scoliosis. Transiliac lengthening remains as an alternative method for achieving balance. If the cause of the postural imbalance is sacral hemiagenesis, a preliminary lumbopelvic fusion is necessary to stabilize the deficient side before transiliac lengthening is performed. The posterior lumbar-iliac percutaneous fusion with CT-guided screws, resulting in a perfect localization of the area involved, it is safe, and it is performed in a short time limiting.
